# Prevalence and morphological and molecular characteristics of *Sarcocystis bertrami* in horses in China

**DOI:** 10.1051/parasite/2019078

**Published:** 2020-01-07

**Authors:** Chun-Li Ma, Yu-Long Ye, Tao Wen, Zhu-Mei Huang, Jing Pan, Jun-Jie Hu, Jian-Ping Tao, Jing-Ling Song

**Affiliations:** 1 School of Biological Sciences, Yunnan University Kunming 650091 PR China; 2 Southeast Asia Biodiversity Research Institute, Chinese Academy of Science Yezin Nay Pyi Taw 05282 Myanmar; 3 College of Veterinary Medicine, Yangzhou University Yangzhou 225009 PR China; 4 Electron Microscope Laboratory, Kunming Medical University Kunming 650500 PR China

**Keywords:** Horse, *Sarcocystis bertrami*, Prevalence, Systematics, Morphology

## Abstract

Three cyst-forming *Sarcocystis* species have been identified in horsemeat; however, there exists considerable confusion concerning their relationships. Here, 74% (34/46) of the examined tissue samples from horses contained sarcocysts based on examination by light microscopy (LM), and the organism was identified as *Sarcocystis bertrami* based on cyst structure. The *S. bertrami* cysts were microscopic (up to 6750 μm in length) and exhibited a striated wall with 2.0–5.1 μm villar protrusions (vps) under LM. Transmission electron microscopy (TEM) observations showed that the vps were tightly packed, similar to “type 11c”. Four genetic markers (18S, 28S, ITS1 and the mitochondrial *cox1* gene) of *S*. *bertrami* were sequenced and analyzed. The 28S and ITS1 sequences are the first records for *Sarcocystis* in horses. The newly obtained sequences of the 18S and *cox1* genes both shared the highest similarities with those of *S*. *bertrami* and *S*. *fayeri* obtained from horses. Phylogenetic analysis based on the 18S, 28S and *cox1* sequences revealed that *S*. *bertrami* and *S. fayeri* formed an independent clade within a group comprising *Sarcocystis* spp. from ruminants and pigs. Therefore, *S*. *bertrami* and *S. fayeri* are considered to represent the same species of *Sarcocystis* in horses, and *S. fayeri* is a junior synonym of *Sarcocystis bertrami*.

## Abstract (fr)

Trois espèces de *Sarcocystis* formant des kystes ont été identifiées dans la viande de cheval, mais il existe une confusion considérable concernant leurs relations. Ici, 74 % (34/46) des échantillons de tissus examinés provenant de chevaux contenaient des sarcocystes selon l’examen par microscopie optique (LM), et l’organisme a été identifié comme *Sarcocystis bertrami* selon la structure du kyste. Les kystes *de S. bertrami* étaient microscopiques (jusqu’à 6 750 μm de longueur) et présentaient une paroi striée avec des saillies villaires (vp) de 2,0 à 5,1 μm sous LM. Les observations en microscopie électronique à transmission ont montré que les vp étaient très serrées, similaires au « type 11c ». Quatre marqueurs génétiques (18S, 28S, ITS1 et le gène mitochondrial *cox1*) de *S. bertrami* ont été séquencés et analysés. Les séquences de 28S et ITS1 sont les premières de *Sarcocystis* chez les chevaux. Les séquences nouvellement obtenues de 18S et des gènes mitochondriaux *cox1* partagent les similitudes les plus élevées avec celles de *S. bertrami* et *S. fayeri* obtenues à partir de chevaux. Une analyse phylogénétique basée sur les séquences de 18S, 28S et cox1 a révélé que *S. bertrami* et *S. fayeri* formaient un clade indépendant au sein d’un groupe comprenant les *Sarcocystis* spp. des ruminants et des porcs. Par conséquent, *S. bertrami* et *S. fayeri* sont considérés comme représentant la même espèce de *Sarcocystis* chez les chevaux, et *S. fayeri* est un synonyme plus récent de *Sarcocystis bertrami*.

## Introduction

Four *Sarcocystis* species have been named in horses (*Equus caballus*) so far: *S. bertrami* Doflein, 1901 [1]; *S. equicanis* Rommel and Geidel, 1975 [22]; *S. fayeri* Dubey, Streitel, Stromberg, and Toussant, 1977 [2]; and *S*. *neurona* Dubey, Davis, Speer, Bowman, de Lahunta, Granstrom, Topper, Hamir, Cummings, and Suter, 1991 [3]. Mature sarcocysts of *S. bertrami*, *S. equicanis* and *S. fayeri* have been found in horses, and all of these species have dogs as their definitive hosts. The horse is considered an aberrant host of *S*. *neurona* because only schizonts have been identified in horses with certainty. There is currently considerable confusion concerning the validity of the three abovementioned cyst-forming *Sarcocystis* species in horses and other equids [4].

Traditionally, sarcocyst structure and life cycle are the two major criteria for naming a new species of *Sarcocystis* in a given intermediate host. However, the morphological characteristics of sarcocysts in horses have been observed to undergo some changes in various stages of development [6, 17]. In the past decade, molecular analysis based on nucleotide sequences has been recommended as a useful and efficient tool for delineating or identifying species of *Sarcocystis* from the same or different hosts [10–12, 14, 15]. The 18S rDNA and mitochondrial *cox1* sequences of *S*. *bertrami* and *S*. *fayeri* have recently been sequenced and deposited in GenBank. However, the relationship of the two species inferred from molecular data is still unclear owing to research work performed and published by different research teams almost simultaneously [19, 23]. There are currently no records of the 28S rDNA and *ITS-1* sequences of *Sarcocystis* spp. from horses available in GenBank.

In the present study, the prevalence of *Sarcocystis* species in horsemeat in China was investigated based on the morphological characteristics of the sarcocysts. Additionally, four genetic markers, 18S rDNA, 28S rDNA, *ITS-1* and mitochondrial *cox1* of the parasite were sequenced and analyzed to augment its descriptions and explore the relationship with *Sarcocystis* spp. in horses.

## Materials and methods

### Morphological examination of sarcocysts from horses

The study protocol was approved by the Animal Ethics Committee of Yunnan University (permission number: AECYU2015021). Horsemeat serves as a food source for humans and is commonly marketed in China. In total, tissues from 46 horses were examined from two abattoirs, one in Kunming City and another in Shilin Prefecture, both of which are located in Yunnan Province, China, from October 2015 to June 2016. From each animal, fresh tissue samples (50 g each) from the esophagus, diaphragm, skeletal muscle, tongue, and heart were examined for sarcocysts. In the laboratory, 40 specimens of approximately 10 × 3 mm in size from each collected sample were pressed and squeezed between two glass slides and then inspected using stereomicroscopy. Thereafter, individual sarcocysts were extracted and isolated from skeletal muscular fibers using needles and processed for light microscopy (LM), transmission electron microscopy (TEM) and DNA analysis.

For TEM, four sarcocysts (two from horse No. 3 and two from horse No. 8) were fixed in 2.5% glutaraldehyde in cacodylate buffer (0.1 M, pH 7.4) at 4 °C, postfixed in 1.0% osmium tetroxide in the same buffer, dehydrated in a graded alcohol series, and embedded in an Epon-Araldite mixture. Ultrathin sections were stained with uranyl acetate and lead citrate and then examined using a JEM100-CX transmission electron microscope (JEOL Ltd., Tokyo, Japan) at 100 kV.

### DNA isolation, PCR amplification, cloning, and sequence analysis

Five individual sarcocysts (cysts 3, 8, 10, 15 and 26) obtained from muscle samples from five horses (Nos. 3, 8, 10, 15 and 26) were subjected to genomic DNA extraction using the phenol/chloroform method after 0.01% proteinase K and 0.25% trypsin digestion. 18S rDNA was amplified with the S1/S4 primers [7]; 28S rDNA was amplified with the KL1/KL3, KL4/KL5b and KL6a/KL2 primers sets [18]; mitochondrial *cox1* was amplified with the SF1/SR9 primers [10, 11]; and *ITS-1* was amplified with the SU1F/5.8SR2 primer pair [12]. The PCR products were purified, cloned, sequenced, and characterized using the methods detailed in a previous paper [14].

## Results

### Observations of sarcocysts of *S*. *bertrami* by LM and TEM

Only sarcocysts resembling those of *S*. *bertrami* were found in 34 of 46 horses (73.9%). The highest prevalence was 63.0% (29/46), recorded in the esophagus, followed by 43.5% (20/46) in skeletal muscle, 23.9% (11/46) in the diaphragm, and 15.2% (7/46) in tongue muscle, whereas none were found in the heart.

**Figure 1:**
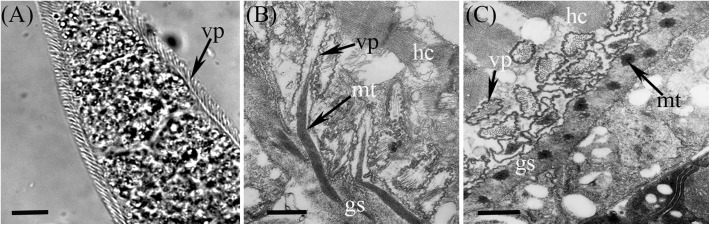
Morphological characteristics of *Sarcocystis bertrami* sarcocysts isolated from the skeletal muscle of a horse. (A) Sarcocyst (unstained, light microscopy) bound by villar protrusions (vps). Scale bar = 10 μm. (B) Longitudinal section of a sarcocyst (under transmission electron microscopy, TEM). The sarcocyst is surrounded by the host cell (hc), and the sarcocyst wall exhibits numerous vps, which are often bent along the cyst surface. The vps contain bundled microtubules (mt) in the core, which penetrate diagonally into the ground substance (gs). Scale bar = 1 μm. (C) Cross-section of a sarcocyst under TEM. Note the bundle of mt in the gs. Scale bar = 1 μm.

Under LM, *S*. *bertrami* sarcocysts were observed to be microscopic, measuring 950–6754 × 65–130 μm (*n* = 30). The sarcocyst wall exhibited numerous 2.0–5.1 μm (*n* = 40)-long villar protrusions (vps) (Fig. 1A). The sarcocysts were septate, and their interior compartments were filled with bradyzoites measuring 14.3–16.1 × 3.1–4.8 μm (*n* = 30) in size.

The ultrastructure of the sarcocysts was similar to “type 11c”. The primary sarcocyst wall exhibited numerous vps, measuring 2.0–4.4 × 0.3–0.8 μm (*n* = 15), which were often tightly packed and folded over the cyst surface (Fig. 1B). The vps contained bundled microtubules in the core, which penetrated diagonally into the ground substance and sometimes reached the interior border of the ground substance (Figs. 1B and 1C). The primary cyst wall presented minute undulations over the entire sarcocyst surface. There was a layer of ground substances of 0.7–1.2 μm (*n* = 12) in thickness immediately beneath the primary sarcocyst wall (Fig. 1C).

### Molecular characterization of 18S rDNA

Genomic DNA was extracted from five individual sarcocysts, and the 18S rDNA, 28S rDNA, mitochondrial *cox1* and *ITS-1* sequences were amplified successfully using their DNA as templates. Four 18S rDNA nucleotide sequences for cysts 3, 8, 10 and 15, two 28S rDNA sequences for cysts 3 and 10, three mitochondrial *cox1* sequences for cysts 3, 8 and 26, and five *ITS-1* sequences for cysts 3, 8, 10, 15 and 26 were successfully assembled, and all of the sequences including the primers were deposited in GenBank.

The four 18S rDNA nucleotide sequences (MH025625–MH025628) were 1592–1594 bp in length and shared an identity of 99.7–99.9% (average 99.8% identity). The most similar sequences in GenBank were those of *S*. *bertrami* from horsemeat in China (KX545397–KX545404, 95.0–100% identity, average 98.0%) and *S. fayeri* from horsemeat from Japan, Canada, and Italy (AB661437–AB661447, AB972440–AB972443 and LC171831–LC171838, 88.9–100% identity, average 96.5% identity).

**Figure 2:**
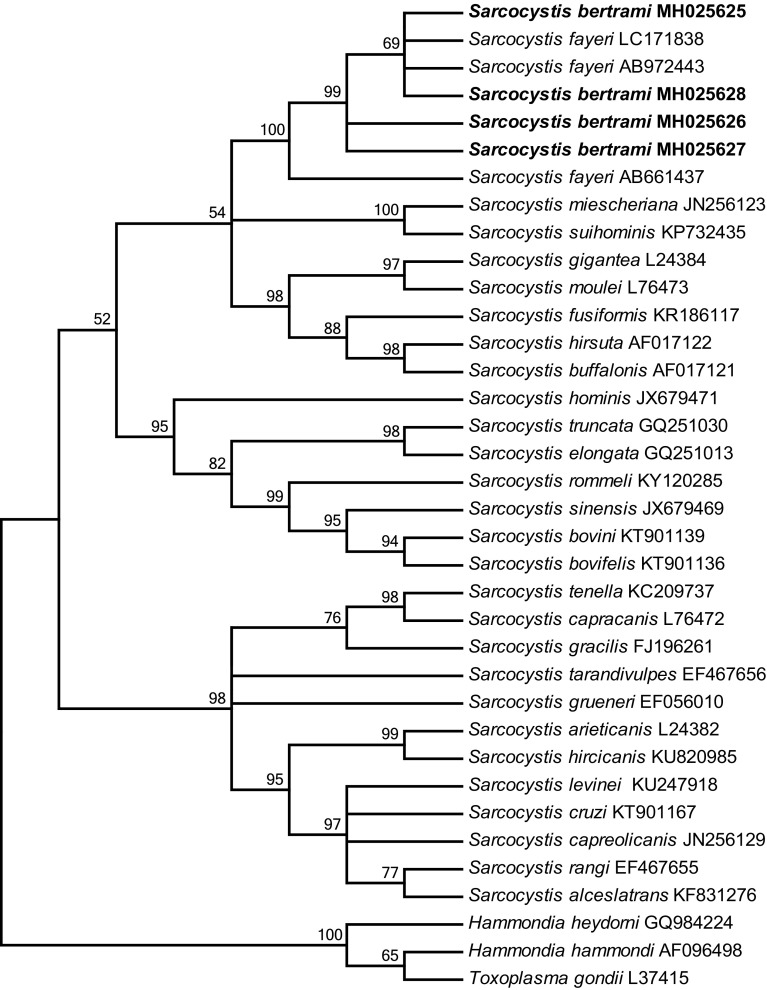
Phylogenetic tree based on 18S rDNA sequences. The tree was built using the maximum parsimony method with the Tree-Bisection-Regrafting algorithm. The analysis involved 36 nucleotide sequences (GenBank accession numbers behind the taxon names) and a total of 1258 positions in the final dataset. The values between the branches represent bootstrap values per 1000 replicates, and values below 50% are not shown. The four new sequences of *Sarcocystis bertrami* (MH025625–MH025628, shown in boldface) formed a clade with *S*. *fayeri* from horses, and the clade was within a group comprising *Sarcocystis* spp. from ruminants and pigs.

The phylogenetic tree inferred from the 18S rDNA sequences revealed that the newly obtained sequences of *S*. *bertrami* formed an individual clade with those of *S*. *fayeri* from horsemeat from Japan (AB661437), Canada (AB972443) and Italy (LC171838) within a group comprising *S. miescheriana* (JN256123) and *S*. *suihominis* (KP732435) from pigs using canids and primates as definitive hosts, respectively, and *Sarcocystis* spp. from ruminants with primates or felids as definitive hosts (Fig. 2).

### Molecular characterization of 28S rDNA

The two 28S rDNA nucleotide sequences (MH025629 and MH025630) were 3456 bp and 3449 bp in length, respectively, and shared 99.2% identity. The most similar sequence in GenBank was that of *S*. *miescheriana* (AF076902) from a pig, but the identity was only 90.4–90.5%.

**Figure 3:**
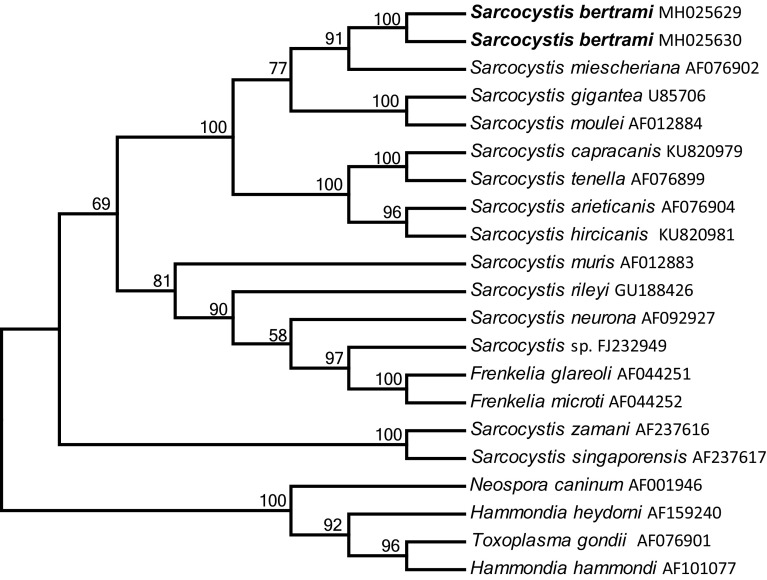
Phylogenetic tree based on 28S rDNA gene sequences. The tree was built using the maximum parsimony method with the Tree-Bisection-Regrafting algorithm. The analysis involved 21 nucleotide sequences (GenBank accession numbers behind the taxon names) and a total of 3680 positions in the final dataset. The values between the branches represent the bootstrap values per 1000 replicates, and values below 50% are not shown. The two new sequences of *Sarcocystis bertrami* (MH025629 and MH025630, shown in boldface) formed a clade with *S. miescheriana* from a pig, and *Sarcocystis* spp. from ruminants.

The phylogenetic tree based on the 28S rDNA sequences revealed that *S*. *bertrami* formed a clade with *S*. *miescheriana* (AF076902), *S*. *gigantea* (U85706) from sheep and *S*. *moulei* (AF012884) from goats, the last two of which use felids as definitive hosts (Fig. 3).

### Molecular characterization of mitochondrial *cox1*

The three mitochondrial *cox1* nucleotide sequences (MH025631–MH025633) were 1060–1063 bp in length and shared an identity of 98.4–99.9% (average 98.9% identity). The most similar sequences in GenBank were those of *S*. *fayeri* (LC171840–LC171857, 98.0–99.8% identity, average 99.0% identity) from horsemeat from Japan, Canada and Italy and *S*. *bertrami* from horsemeat from China (KY399751–KY399755, KY399758, KY399760–KY399762, MF152616–MF152619, 98.1–99.2% identity, average 98.8% identity).

**Figure 4:**
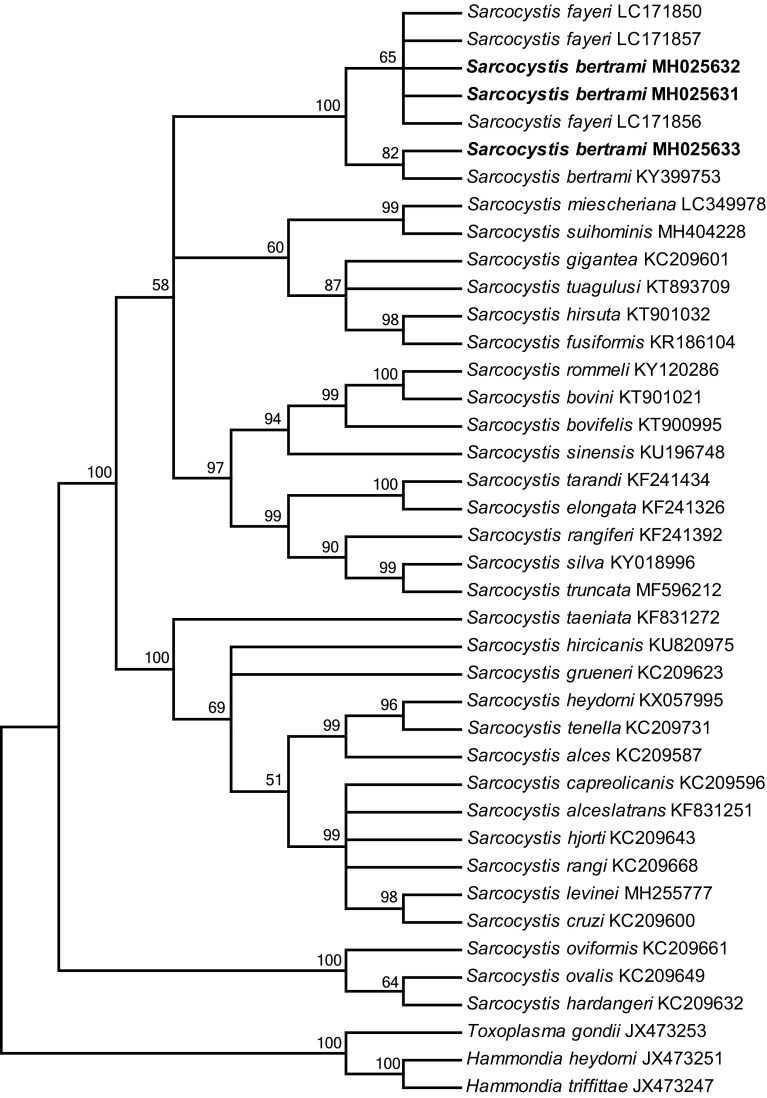
Phylogenetic tree based on mitochondrial *cox1* sequences. The tree was built using the maximum parsimony method with the Tree-Bisection-Regrafting algorithm. The analysis involved 40 nucleotide sequences (GenBank accession numbers behind the taxon names) and a total of 936 positions in the final dataset. The values between the branches represent the bootstrap values per 1000 replicates, and values below 50% are not shown. The three new sequences of *Sarcocystis bertrami* (MH025631–MH025633 shown in boldface) formed a clade with *S*. *bertrami* and *S*. *fayeri* from horses within a group comprising *Sarcocysti*s spp. from pigs and ruminants.

The phylogenetic tree based on the mitochondrial *cox1* sequences revealed that the newly obtained sequences of *S*. *bertrami* formed an individual clade with those of *S*. *bertrami* from horsemeat from China (KY399753) and *S*. *fayeri* from horsemeat from Japan (LC171856), Canada (LC171850) and Italy (LC171857) within a group comprising *S. miescheriana* (LC349978), *S*. *suihominis* (MH404228), and *Sarcocystis* spp. from ruminants with felids as known or suspected definitive hosts (Fig. 4).

### Molecular characterization of *ITS-1*

The five *ITS-1* nucleotide sequences (MH025634–MH25638) were 934–936 bp in length and shared an identity of 96.7–98.3% (average 97.4% identity). BLAST searches using only the *ITS-1* region sequences of approximately 660 bp from *S*. *bertrami* revealed that no sequences in GenBank shared significant similarities with them.

## Discussion

*Sarcocystis* is a common parasitic protozoan with a worldwide distribution that is commonly found in a variety of mammals and birds, especially common in domesticated food animals. In the current study, the prevalence of sarcocysts in Chinese horses was 73.9% (34/46), and it has been reported to be higher than 68.8% (22/32) in Turkish horses [21], 46.2% (55/119) in Moroccan horses [16], 62.2% (245/349) in British horses [5], and 13.2% (12/91) in Belgian horses [8], but lower than 93.0% (40/43) in Mongolian horses [9].

To date, three *Sarcocystis* species, *S*. *bertrami*, *S*. *equicanis* and *S*. *fayeri*, have been identified from horsemeat, all of which have the dog as the definitive host. However, there is considerable confusion concerning the validity of the above species due to their similar life cycles and morphology. On the basis of TEM morphology, Dubey et al. (2016) suggested that there are two valid species of *Sarcocystis* in horses: a thick-walled species (*S*. *fayeri*), with a “type 11a” sarcocyst wall, and a thin-walled species (*S*. *bertrami* synonym *S*. *equicanis*), with a “type 11c” sarcocyst wall. The critical distinction between the two sarcocyst wall types is that “type 11a” exhibits nearly upright vps, but “type 11c” exhibits packed and folded vps [4]. The sarcocysts reported here were diagnosed as *S*. *bertrami* based on their similarity with “type 11c”, which has been demonstrated previously for *S*. *equicanis* from European horses [13], for *Sarcocystis* sp. from Mongolian horses [20] and for *S*. *bertrami* from Chinese horses [23].

Nucleotide sequence analysis has proven to be a useful tool for delineating or identifying species of *Sarcocystis* from the same or different hosts, and different genetic markers have shown different levels of intra- or inter-specific sequence diversity [14, 15]. There are only sequences of 18S rDNA and mitochondrial *cox1* from *S*. *bertrami* and *S*. *fayeri* currently deposited in GenBank. In our analysis, the newly obtained 18S rDNA sequences exhibited up to 100% identity (average 98.0% and 96.5% identity, respectively) with those of *S*. *bertrami* and *S*. *fayeri* from horses provided in GenBank; the newly obtained mitochondrial *cox1* sequences shared the highest identity of 98.0–99.8% (average 99.0%) with those of *S*. *fayeri*, followed by *S*. *bertrami* (98.1–99.2% identity, average 98.7% identity). A possible explanation for the high similarities of the two parasites is that both represent the same species of *Sarcocystis* in horses. The protrusions observed via TEM may appear to be upright or folded depending on the plane of the cut section. Phylogenetic analysis based on the 18S rDNA, 28S rDNA and mitochondrial *cox1* sequences also confirmed the close relationship between *S*. *bertrami* and *S*. *fayeri*.

In conclusion, we found a high prevalence rate of *Sarcocystis* in horses in China, and only *S*. *bertrami* was identified based on the cyst ultrastructure. Based on 18S rDNA and mitochondrial *cox1*, *S*. *bertrami* and *S*. *fayeri* are inferred to represent the same species from horses. According to the general rules of the International Code of Zoological Nomenclature, *S*. *fayeri* should be considered a junior synonym of *S*. *bertrami*.

## Conflict of interest

The authors declare that they have no competing interests.
